# LDB1-mediated transcriptional complexes are sensitive to islet stress

**DOI:** 10.1080/19382014.2021.2016028

**Published:** 2021-12-30

**Authors:** Yanping Liu, Jessica D. Kepple, Anath Shalev, Chad S. Hunter

**Affiliations:** a Department of Medicine, Division of Endocrinology Diabetes and Metabolism University of Alabama at Birmingham, Birmingham, AL, USA; b Comprehensive Diabetes Center, University of Alabama at Birmingham, Birmingham, AL, USA

**Keywords:** Transcription factor, co-regulator, diabetes, pancreas, islet, glucose, cytokine, palmitate

## Abstract

Excess nutrients and proinflammatory cytokines impart stresses on pancreatic islet β-cells that, if unchecked, can lead to cellular dysfunction and/or death. Among these stress-induced effects is loss of key β-cell transcriptional regulator mRNA and protein levels required for β-cell function. Previously, our lab and others reported that LIM-domain complexes comprised the LDB1 transcriptional co-regulator and Islet-1 (ISL1) transcription factor are required for islet β-cell development, maturation, and function. The LDB1:ISL1 complex directly occupies and regulates key β-cell genes, including *MafA, Pdx1*, and *Slc2a2*, to maintain β-cell identity and function. Given the importance of LDB1:ISL1 complexes, we hypothesized that LDB1 and/or ISL1 levels, like other transcriptional regulators, are sensitive to β-cell nutrient and cytokine stresses, likely contributing to β-cell (dys)function under various stimuli. We tested this by treating β-cell lines or primary mouse islets with elevating glucose concentrations, palmitate, or a cytokine cocktail of IL-1β, TNFα, and IFNγ. We indeed observed that LDB1 mRNA and/or protein levels were reduced upon palmitate and cytokine (cocktail or singly) incubation. Conversely, acute high glucose treatment of β-cells did not impair LDB1 or ISL1 levels, but increased LDB1:ISL1 interactions. These observations suggest that LDB1:ISL1 complex formation is sensitive to β-cell stresses and that targeting and/or stabilizing this complex may rescue lost β-cell gene expression to preserve cellular function.

## Introduction

The insulin-producing β-cell, tasked with mediating glucose homeostasis via glucose-stimulated insulin secretion (GSIS), is central to the etiology of type 1 (T1D, loss of pancreatic β-cells) or type 2 diabetes (T2D, reduced functional β-cell mass).^1–4^ β-cells are exquisitely tuned to the milieu of external stimuli to modulate insulin expression, production, maturation, and secretion. While acute elevations in plasma glucose levels impart stimulatory effects of increased *Insulin* gene transcription and hormone secretion, prolonged supraphysiological glucose treatment in cell lines or *in vivo* (i.e., glucotoxicity) reduces β-cell function and viability. These effects are mediated, in part, by changes in levels and/or function of various islet transcription factors (TF). One example is the TF MAFA, which is sensitive to changes in nutrients and cytokines. MAFA expression and function is required for *Insulin* transcription and is induced by acute glucose treatment.^5–9^ However, β-cell stresses imparted by prolonged *in vitro* incubation with high glucose, fatty acids, or proinflammatory cytokines (e.g., TNFα, IFNγ, Interleukin-1β (IL-1β);^10–12^), as well as *in vivo* diabetic mouse models (e.g., *db*/*db*,^13,14^), leads to MAFA inactivation and loss, likely stimulating a cascade of β-cell dysfunction.^15^

Similarly, another critical TF PDX1, required for β-cell function and identity, is differentially regulated by external stimuli. The expression of *Pdx1* mRNA *in vitro* is negatively impacted by prolonged exposure to high glucose.^10,16^ Additionally, PDX1 binding to the target *MafA* Region 3 control domain was reduced under oxidative stress imparted by H_2_O_2_ treatment.^13^ Importantly, both MAFA and PDX1 (as well as the TF NKX6.1) protein levels were lost from human T2D islet β-cells, thus linking these TF levels to human β-cell dysfunction and diabetes.^13^

Our lab and others have shown that the LIM-homeodomain transcription factor, Islet-1 (ISL1), and interacting co-regulator, LIM domain-binding protein 1 (LDB1), are required for β-cell development and function.^17–20^ Comparative tissue- and cell-type-specific knockout mouse models revealed that LDB1:ISL1-containing complexes are necessary for β-cell development, identity, survival, and insulin secretory function via direct regulation of several key β-cell gene targets, including *MafA, Pdx1, Slc2a2, Glp1r*, among others.^17–20^ Previously published work from our lab utilizing *in vitro* protein interaction screens revealed the Single-Stranded DNA-Binding protein 3 (SSBP3, also called SSDP1^21,22^) co-regulator participates in β-cell LDB1:ISL1 complexes and contributes to the regulation of *MafA* expression in β-cells.^23^ Further, ISL1 and LDB1 are maintained in human islets,^19,20^ highlighting the conservation and importance of these factors to mammalian β-cells and glucose homeostasis. However, little is known of whether the expression and/or interactions of ISL1 and LDB1 are modulated by β-cell stimuli or stressors.

In this study, we surveyed mouse β-cell lines and islets treated with low and high glucose, palmitate, or proinflammatory cytokines to assess the effects on ISL1 and/or LDB1 mRNA and protein levels as well as protein–protein interactions. Our findings show that at least LDB1 experiences specific reductions in mRNA and protein under certain stimuli, including palmitate and cytokine treatments, and that LDB1:ISL1 protein interactions are stimulated under high glucose conditions, yet reduced by cytokine treatment. This further supports that the LIM-domain complex of the ISL1 TF and LDB1 co-regulator are central to β-cell mediated glucose homeostasis.

## Materials and methods

### Islet isolation and treatments

Islets were isolated from wild-type C57BL/6J mice by handpicking after collagenase P (Roche, 11249002001) digestion, as described.^24^ Islets were recovered overnight in RPMI 1640 media (Gibco, 11875–093) supplemented with 10% Fetal Bovine Serum and 1% Penicillin/Streptomycin (GIBCO) prior to incubation with various cell stimuli described below. To examine glucose effects, islets or cell lines (mouse Min6, rat Ins-1) were incubated for 4 hours (hr) or overnight (~12-16 hr) with increasing concentrations of glucose in RPMI 1640 (3.9, 7.8, 15.6, and 31.1 mM), then qPCR experiments performed. For testing cytokine effects, in a separate pool of rodent islets or cells, TNFα (R&D Systems #410-MT, 10 ng/mL), IFNγ (R&D Systems #485-MI, 100 ng/mL), and/or IL-1β (R&D Systems # 401-ML, 5 ng/mL) cytokines were applied singly or as a cocktail for 4 hr, as compared to PBS vehicle control. Finally, to test impacts of palmitate, independent preparations of mouse islets and cells were treated with control BSA or palmitic acid (0.5 mM, prepared as described in ref.^25^) for 4 hr to overnight. After all treatments, islets and cells were collected and protein or RNA were extracted for downstream analyses. All treatments were performed with independent islet and cell line preparations at least three times.

### Human islets

As detailed in a prior study,^26,27^ donor human islets were treated with 50 U/mL (0.36 ng/mL) hIL-1β, 1000 U/mL (13.16 ng/mL) hTNFα, 1000 U/mL (50 ng/mL) hIFNγ cytokine cocktail (R&D Systems) or control DMSO for 24 hr, then RNA was isolated. Here, the cDNA was re-analyzed by qPCR (detailed below) for *MAFA, PDX1, NKX6.1, LDB1*, and *ISL1* levels.

### Western blotting

After glucose, palmitate or cytokine treatment, as described for mouse islets above, Ins-1 protein extracts^23^ were separated by 10% SDS-PAGE (Bio-Rad) then transferred to a polyvinylidene difluoride (PVDF, Bio-Rad) membrane. For Western blotting (WB), membranes were blocked in PBS/Tween plus 5% nonfat dry milk for 1 hr, followed by incubation with α-LDB1 (sc-11198x, 1:1000; Santa Cruz Biotechnology, Inc), α-MAFA (1:1000, NBP1–00121, Novus), α-ISL1 (1:1000, 39.4D5-c; Developmental Studies Hybridoma Bank), or α-NKX6.1 (1:1000, F55A10-c; Developmental Studies Hybridoma Bank) antibodies overnight at 4°C. A rabbit β-Actin antibody was used for loading control (#4967S, Cell Signaling Technology). Western blot primary antibody information can be found in Supplemental Table 3. The PVDF membrane was washed and incubated with species-matched horseradish peroxidase-conjugated secondary antibodies (Promega or Santa Cruz Biotechnology) followed by addition of Luminata Forte substrate (Millipore) and visualized using Chemidox XRS + Imager (Bio-Rad). All WB experiments were performed with at least three different cell preparations.

### Proximity ligation assay (PLA)

PLA was performed using glucose-, cytokine-, or palmitate- (or control) treated Min6 cells (as described above for primary mouse islets) using the Duolink In Situ Red Mouse/Rabbit kit per manufacturer’s instructions (Millipore/Sigma, DUO92101), and as described.^28^ Briefly, Min6 cells were seeded into multi-well chambered cell culture slides (354114, Corning) prior to treatment. After stimuli treatment for 4 hr in low (2.5 mM) or high (25 mM) glucose, the cells were formaldehyde fixed and then incubated with rabbit α-LDB1 (kind gift from Dr. Paul Love, NIH) and mouse α-ISL1 antibodies (39.4D5-c, Developmental Studies Hybridoma Bank) prior to PLA procedure. Cell nuclei were counterstained with DAPI, and then fluorescent signals were visualized by a Zeiss LSM710 confocal microscope and images processed by Zen software (Zeiss). To quantify PLA interactions, blinded images were quantified for the ratio of nuclei lacking PLA interaction signals over total nuclei, as described.^28^ PLA staining and quantification were performed on three separate occasions. PLA primary antibody information can be found in Supplemental Table 4.

### siRNA transient transfection into mouse islets

For small interfering RNA (siRNA) transfection experiments, primary mouse islets were dispersed into single cells as described,^23,29^ then seeded in 6- or 12-well plates and transfected with 50 nM On-Target Plus Smart Pool targeting *Ldb1* (*siLdb1*, L-043882-00-0005 GE Healthcare/Dharmacon/Horizon) or nontargeted scrambled control (*siSCR*, D-001810-10-05 GE Healthcare/Dharmacon/Horizon) using RNAiMax (13778030; Life Technologies). mRNA analyses were performed 48 hr post-transfection.

### Quantitative RT-PCR (qPCR)

RNA was isolated from primary mouse islets or mouse Min6 cells using the RNeasy Mini Plus kit (74134; Qiagen) and cDNA was made using the iScript cDNA synthesis kit (170–8840; Bio-Rad). qPCR reactions were performed using iTaq SYBR Green (172–5124; Bio-Rad) in duplicate using a LightCycler 480 II (Roche) and analyzed using the 2^ΔΔCT^ method, with normalization to the *36B4, 18S*, or *Gapdh* housekeeping genes. Primer sequences can be found in Supplemental Tables 1–2.

### Statistical analyses

Data are presented as mean ± SEM. Significance was determined after performing a Student’s t-test or Analysis of Variance (ANOVA) with Dunnett’s testing for multiple comparisons, as appropriate, for which *P* < 0.05.

## Results

### Glucose levels modulate β-cell enriched mRNAs, including *Isl1*

**Figure 1.**
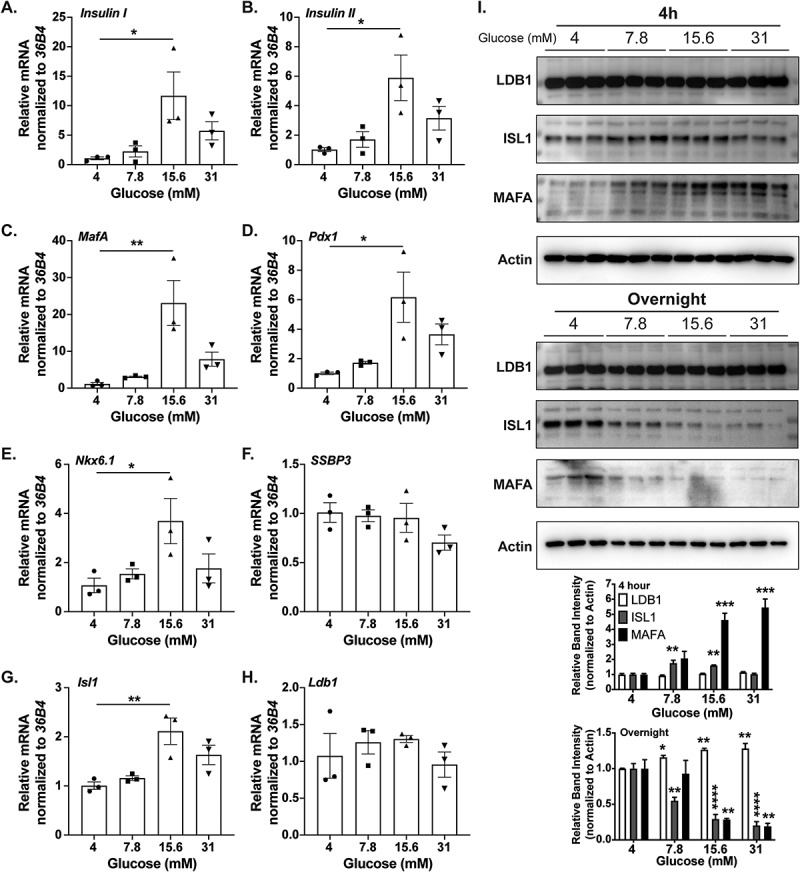
Glucose levels modulate β-cell enriched mRNAs, including *Isl1*. A-H. Relative mRNA quantification of various β-cell factors (*Insulin I, Insulin II, MafA, Pdx1, Nkx6.1, SSBP3, Isl1*, and *Ldb1* respectively) from primary mouse islets after overnight culturing in media with increasing glucose concentrations (4 mM, 7.8 mM, 15.6 mM, and 31 mM). Low glucose (4 mM) was set as onefold, and *36B4* was used as the housekeeping gene; n = 3 for each treatment group. I. Top: Western blotting of Ins-1 cell extracts for LDB1, ISL1, or MAFA after 4 hr or overnight treatment with similar glucose concentrations as above. Actin was included as a loading control. Bottom: Densitometry quantification of 4 h or overnight treated LDB1, ISL1, or MAFA Western blot protein levels, normalized to Actin. *, *P* < .05; **, *P* < .01; ***, *P* < .001; ****, *P* < .0001 based on one-way ANOVA.

To determine the effects of glucose levels on LIM-factor complex members and regulated gene targets, we employed primary wild-type C57BL/6J mouse islets incubated overnight with increasing glucose concentrations, from 4 mM to 7.8 mM, 15.6 mM, and 31 mM. Stimulation of islets with 15.6 mM glucose resulted in a significant increase in mRNAs encoding *Insulin I/II* and the β-cell identity transcription factors *Mafa, Pdx1*, and *Nkx6.1* (Figure 1A-E), required for β-cell responses.^9,13^ Interestingly, we found differential effects of glucose on the mRNA of LIM-complex associated factors. The *Isl1* TF mRNA was significantly elevated after stimulation with 15.6 mM glucose while mRNA encoding the co-regulators *SSBP3* and *Ldb1* were unchanged (Figure 1F-H). Utilizing a rat Ins-1 cell line, we confirmed that LDB1 co-regulator protein levels were unchanged in response to elevating concentrations of glucose (Figure 1I). Moreover, ISL1 protein levels appeared increased (when compared to basal 4 mM glucose levels) upon 4 hr incubation of the Ins-1 cells with 7.8 mM and 15.6 mM glucose, whereas prolonged (i.e., overnight) incubation in increasing glucose levels resulted in a reduction of ISL1 protein. Similarly, MAFA protein was elevated upon increasing glucose levels during a 4 hr treatment, which was ablated with the prolonged glucose culturing, an observation that is consistent with known MAFA responses to glucose stimulation. Overall, these data suggest that at least ISL1 mRNA and protein levels are sensitive to changes in glucose and therefore may contribute to the β-cell transcriptional responses to glucose, such as *MafA* expression.

### Palmitate treatment downregulates LDB1 and SSBP3 co-regulator

**Figure 2.**
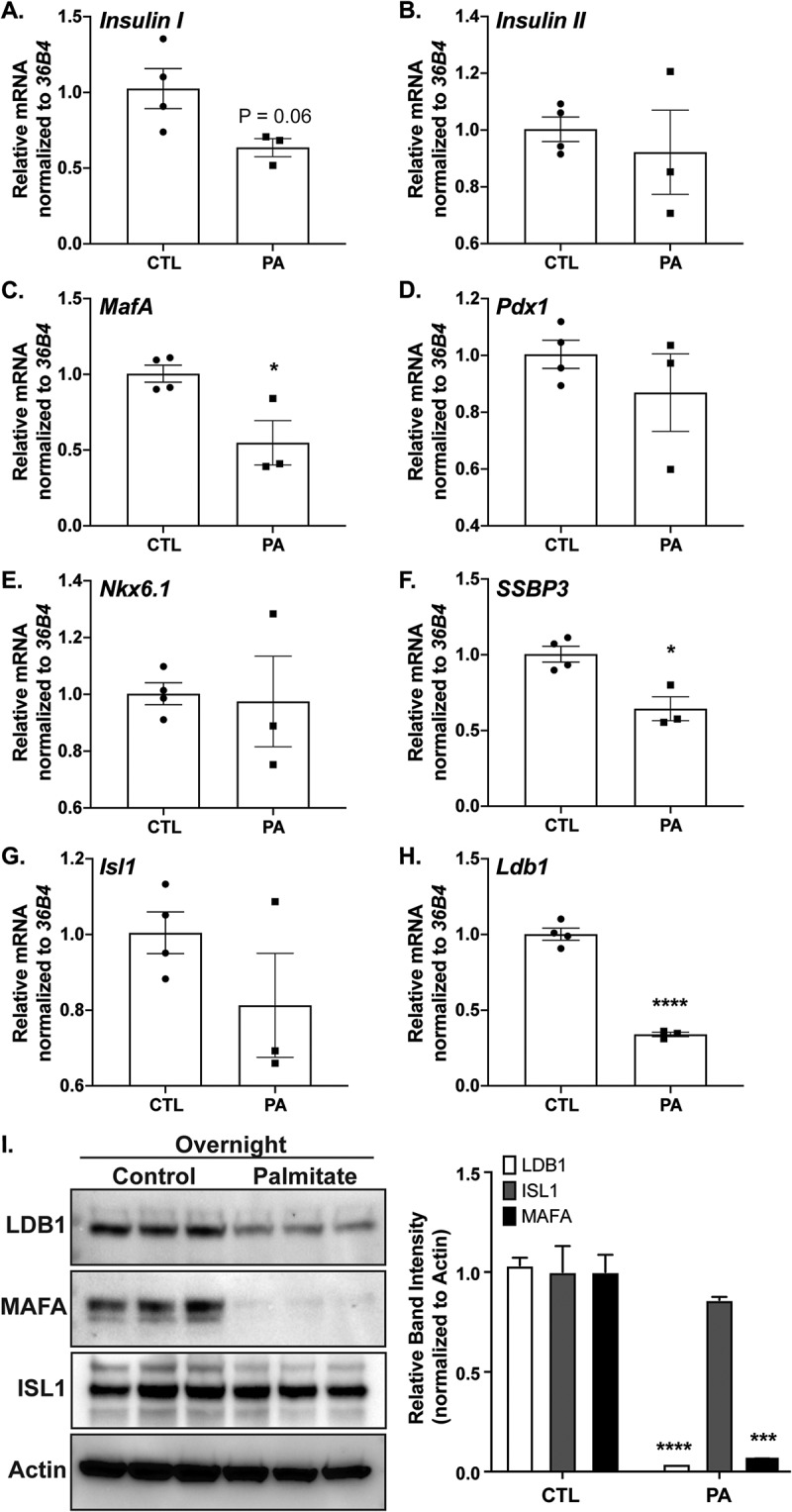
Palmitate treatment downregulates LDB1 and SSBP3 co-regulator levels. A-H. Relative mRNA quantification of various β-cell factors (*Insulin I, Insulin II, MafA, Pdx1, Nkx6.1, SSBP3, Isl1*, and *Ldb1*, respectively) from primary mouse islets after overnight culturing in media with control BSA (set as 1-fold) or palmitic acid (PA, 0.5 mM). *36B4* was used as the housekeeping gene; n = 3 for each treatment group. **I**. Left: Western blot of LDB1, MAFA, and ISL1 in Ins-1 cell extracts after overnight treatment with BSA control or 0.5 mM PA. Actin was included as loading control. Right: Densitometry quantification of LDB1, ISL1, or MAFA Western blot protein levels, normalized to Actin. *, *P* < .05; ***, *P* < .001; ****, *P* < .0001, based on Student’s t-test.

Elevated plasma fatty acids are associated with β-cell dysfunction and impaired insulin secretion.^30,31^ We set out to examine if overnight palmitate (palmitic acid, PA) treatment of primary mouse islets or rat Ins-1 cells impacts LIM-domain transcriptional complex components ISL1, LDB1, and SSBP3 and regulated gene targets.^18–20,23^ Assessment of BSA control- or PA-treated islets via qPCR revealed LIM complex-regulated *Insulin I* and *MafA* mRNA levels^18,19,32^ were reduced (trending or significantly, respectively) upon PA treatment while *Insulin II, Pdx1*, and *Nkx6.1* were unaffected (Figure 2A-E). Interestingly, while the mRNA encoding TF *Isl1* was also unaffected, *Ldb1* and *SSBP3* mRNA were significantly reduced after PA treatment (Figure 2F-H). Further, in overnight PA-treated Ins-1 cells, we also observed overt reductions of LDB1 protein, in addition to a severe loss of MAFA protein,^32^ consistent with mRNA changes (Figure 2I). This suggests that LIM complexes containing the LDB1 and SSBP3 co-regulators are sensitive to fatty acid treatment and may contribute to altered *MafA* expression under lipotoxic β-cell stress conditions.

### Critical β-cell factors are reduced upon cytokine treatment in mouse and human islets

**Figure 3.**
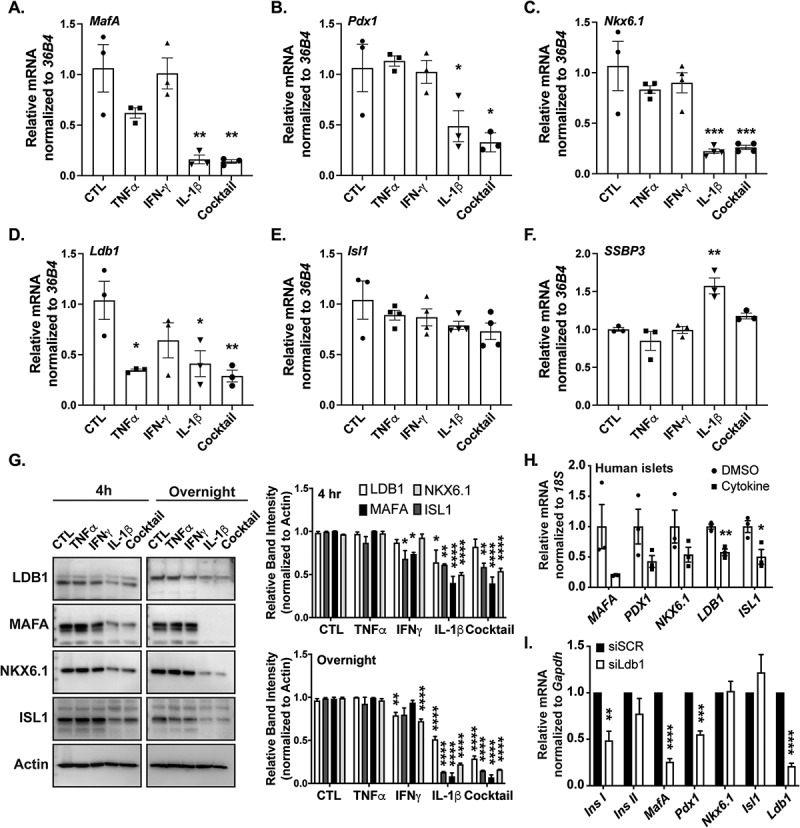
Critical β-cell factors are reduced upon cytokine treatment in mouse and human islets. A-F. Relative mRNA quantification of various β-cell transcriptional regulator genes (*MafA, Pdx1, Nkx6.1, Ldb1, Isl1*, and *SSBP3*, respectively) from primary mouse islets after 4 h culturing with single cytokines (Tnfα, Ifnγ, IL-1β) or a cocktail of each, as compared to PBS vehicle controls (set to 1-fold). *36B4* was used as the housekeeping gene; n = 3–4 for each treatment group. G. Left: LDB1, MAFA, NKX6.1, and ISL1 Western blot with Ins-1 cell extracts after 4 h or overnight treatment with single cytokines, or a cocktail, as compared to control. Actin was included as loading control. Right: Densitometry quantification of 4 h or overnight treated LDB1, MAFA, NKX6.1, or ISL1 Western blot protein levels, normalized to Actin. H. Human islets were treated with a DMSO vehicle or a cytokine cocktail,^26,27^ then mRNA measured. *18S* was used as the housekeeping gene; n = 3. I. Quantification of β-cell mRNAs from primary mouse islets after siRNA-mediated *Ldb1* knockdown using *Gapdh* as the housekeeping gene; n = 3. *, *P* < .05; **, *P* < .01; ***, *P* < .001; ****, *P* < .0001 based on one-way ANOVA or Student’s t-test.

As diabetes is linked to cytokine-mediated inflammation leading to loss of functional β-cell mass, we next asked whether treating islets with three key cytokines (TNFα, IFNγ, IL-1β, or a cocktail of all three) affects LIM-domain complex factor expression levels. We treated isolated primary mouse islets with the various cytokine combinations (or vehicle control) for 4 hr prior to isolating RNA for qPCR assessment. Cytokine cocktail and IL-1β treatment alone led to significant decrements in *MafA, Pdx1*, and *Nkx6.1* TF mRNAs (Figure 3A-C). Interestingly, although there were no reductions of *Isl1* or *SSBP3* mRNA with cytokine treatment, *Ldb1* mRNA was significantly reduced upon incubation with TNFα, IL-1β, and the cocktail, with a trending reduction with IFNγ (Figure 3D-F). The effects of cytokines on β-cell proteins were confirmed with extracts from cytokine treated Ins-1 cells. Consistent with mRNA changes, MAFA and NKX6.1 proteins were reduced upon 4 hr and overnight treatment with either IL-1β or the cytokine cocktail (Figure 3G). After 4 hr, IL-1β also imparted a reduction of LDB1. With the overnight-treated Ins-1 cells, LDB1 was significantly reduced with IFNγ, IL-1β, and cocktail treatment (Figure 3G). We also measured various TF mRNA levels in human islet samples treated with cytokine cocktail as described previously.^26^ Indeed, *ISL1* and *LDB1* were significantly reduced in the cytokine-treated human islets, with a trend toward reduction in *MAFA, PDX1*, and *NKX6.1* (Figure 3H). To correlate downregulation of *Ldb1* mRNA with target genes also impacted by cytokine treatments (e.g., *MafA*), we performed an siRNA *Ldb1* knockdown (*siLdb1*) in dispersed primary mouse islet cells without cytokines, as described in our past studies.^23,33^ *SiLdb1* transfection resulted in an approximately 80% reduction of *Ldb1* mRNA, consistent with levels of *Ldb1* loss upon cytokine cocktail treatment (Figure 3D, I). Loss of *Ldb1* resulted in reductions in *Ins1, MafA* and *Pdx1*, known targets of LDB1:ISL1 transcriptional complexes (Figure 3I).^17–20^ Overall, these data suggest that LDB1 is a cytokine-sensitive target, which in turn may contribute to *MafA* and/or *Pdx1* loss under proinflammatory cytokine conditions.

### Glucose and cytokines regulate LDB1:ISL1 protein interactions in Min6 β-cells

**Figure 4.**
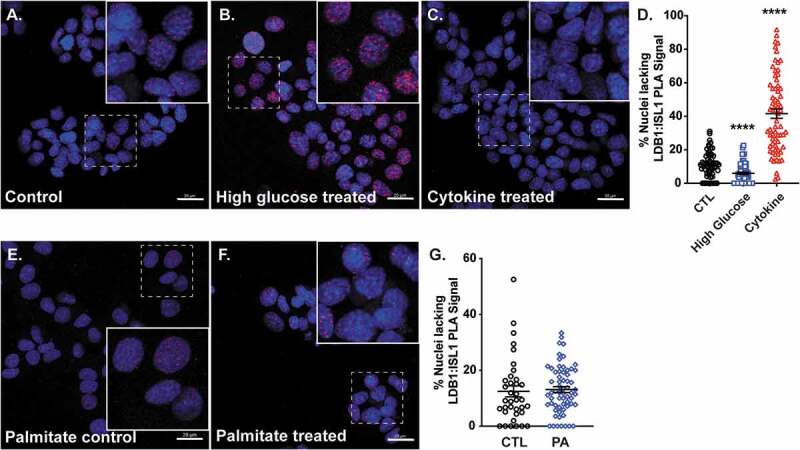
Glucose and cytokines regulate LDB1:ISL1 protein interactions in Min6 β-cells. A-C. PLA immunofluorescence (red focal spots) and DAPI nuclear signals (blue) in Min6 cells treated with (A) control low glucose, (B) high-glucose, and (C) TNFα/IL-1β/IFNγ cytokine cocktail. Insets are 200% magnified images to show greater detail, which are taken from the main field, denoted by dashed white boxes. D. Quantification of nuclei lacking at least 1–2 nuclear PLA signals (red focal spots) in Min6 cells in A-C. E-F. PLA immunofluorescence (red) and DAPI nuclear signals (blue) in Min6 cells treated with (E.) Palmitate control-BSA or (F.) palmitic acid (PA). G. Quantification of nuclei lacking at least 1–2 nuclear PLA signals (focal spots) in Min6 cells in E-F. n = 3; scale bar = 20 μm. ****, *P* < .0001 based on Student’s t-test.

Given that the various β-cell stimuli applied above appeared to modulate ISL1 and/or LDB1 protein or mRNA levels, we next asked whether LDB1 and ISL1 interactions were altered under these conditions. To this end, we cultured mouse Min6 cells for 4 hr in either low and high glucose, cytokine cocktail, or with palmitate and then assessed interactions *in situ* by the proximity ligation assay (PLA,^28^). Changes in nuclear interactions were quantified by manually counting the number of cell nuclei that *lacked* PLA signals, with a higher nuclei count suggesting LDB1:ISL1 interactions were lost, while a lower nuclei count indicates that interactions were elevated. Compared to low glucose control cells (2.5 mM, Figure 4A), high glucose treated cells (25 mM) had significantly greater LDB1:ISL1 PLA interactions (Figure 4B, D), while cytokine treatment resulted in a loss of interactions in Min6 nuclei (Figure 4C-D). Conversely, PA treatment had no significant effect on LDB1:ISL1 interactions when compared to BSA-treated controls (Figure 4E-G), despite significant effects on LDB1 mRNA and protein levels (Figure 2H, I). These data suggest that elevated LDB1:ISL1 associations in response to acute elevation in glucose may participate in β-cell transcriptional responses, while elevated cytokines diminish the accumulation of this complex.

## Discussion

This study aimed to assess how LIM-transcriptional complexes, in part comprised the ISL1 TF and interacting co-regulator SSBP3 and LDB1, are impacted by various β-cell stimuli and stressors. Our goal was to evaluate how treatments of glucose, fatty acids, or cytokines modulate the expression and interactions of LIM components and downstream islet TFs, including MAFA and PDX1.^10,13,16,32^ *Acute* treatment of primary islets or β-cell lines allowed us to focus our analysis on short-term dynamic responses in complex formation. In addition, since ISL1 and LDB1 are critical for adult β-cell identity and function, via regulation of the β-cell transcriptome, including targets *MafA, Pdx1*, and *Slc2a2* (encoding the GLUT2 glucose transporter),^17–20^ findings from this study could be especially informative. Thus, defining the impacts of the diabetic milieu, whether T1D-associated pro-inflammatory cytokines or T2D-associated hyperglycemia and lipotoxicity, has the promise to reveal how β-cell transcriptional profiles may be altered upon changes in expression of LDB1:ISL1 complex components and/or complex formation.

Principally, we found that the LDB1 co-regulator mRNA and protein were reduced by palmitate or cytokine treatment of primary mouse islets or β-cell lines. Conversely, high-glucose had little effect on *Ldb1* or *SSBP3* levels, yet *Isl1* (as well as *Insulin I*/*II, MafA, Pdx1*, and *Nkx6.1*) mRNA was elevated at stimulatory glucose (15.6 mM), supporting the origins of ISL1 discovery as an *Insulin* promoter binding factor.^34^ Strikingly, PLA analyses in Min6 cells revealed increased LDB1:ISL1 interactions with high-glucose, diminished interactions with cytokine treatment, and unchanged interactions with palmitate.

Several key findings are evident from this study. First, we found LDB1 and ISL1 have greater protein interactions upon glucose stimulation. This is similar to published observations that interactions between PDX1 and components of the SWI/SNF co-regulatory complex also were elevated in mouse islets after glucose treatment.^28^ This suggests that, in addition to the necessary roles in β-cell identity *in vivo*, LDB1:ISL1 complexes are critical for acute β-cell responses to glucose, likely including the *trans*-activation of target genes. Several questions remain, including the specificity of this interaction in response to glucose. Are LDB1:ISL1 complexes uniquely responsive to glucose, or might we also observe greater interactions between other LDB1 or ISL1 interactors (e.g., SSBP3 and/or Rnf20/Rnf40^23,33^)? The fact that the PLA assay revealed no change in LDB1:ISL1 interactions in response to PA treatment despite PA causing downregulation of LDB1 mRNA and protein levels may be due to sufficient LDB1 protein remaining in the PA-treated cells to preserve ISL1 associations, whereas perhaps interactions with other LDB1 partners (e.g., SSBP3) were lost. Additionally, given the striking results from the PLA in the Min6 cell line, we are actively extending these studies to primary mouse and human islets to examine if these observations are found in primary islets. Another key finding from this work was the apparent sensitivity of *Ldb1* mRNA levels to β-cell treatment with multiple cytokines (i.e., TNFα, IL-1β, and the cocktail). This contrasts with *MafA, Pdx1*, and *Nkx6.1*, which were exclusively affected by IL-1β and cocktail treatment (and thus likely NF-κB signaling^12^). Prior studies also found MAFA expression and/or activity to be negatively regulated by IL-1β.^11,12^ Collectively, these data suggest that LDB1-mediated transcriptional complexes are negatively targeted by cytokine signaling in β-cells, and thus represent a potential entry point for therapeutics that prolong β-cell function and/or survival during a pro-inflammatory attack. Interestingly, after a siRNA-mediated *Ldb1* knockdown in dispersed mouse islets, mRNA analysis revealed reduced *MafA, Ins I*, and *Pdx1*, similar to mouse and human islets cultured with cytokines. These data suggest that inhibitory effects on gene expression in cytokine-treated β-cells may be due in part to LDB1 loss. Therefore, forced over-expression or stabilizing LDB1:ISL1 complexes in stressed β-cells may help maintain gene expression and function. Future efforts will also aim to identify the *cis*- and *trans*-regulatory elements in the *Ldb1* locus that are responsible for modulating levels of this co-regulator in the β-cell, as knowledge gained may inform strategies for maintaining expression (and thus, the transcriptional complex with ISL1) in the face of inflammation or other β-cell stressors.

## Supplementary Material

Supplemental Material

## Abbreviations

LIM = Lin11, ISL1, Mec3; ISL1 = Islet-1; LDB1 = LIM-domain binding protein 1; SSBP3 = Single Stranded DNA-Binding Protein 3; T1D = Type 1 diabetes; T2D = Type 2 diabetes; GSIS = glucose-stimulated insulin secretion
