# Supplementary material for: LDB1-mediated transcriptional complexes are sensitive to islet stress
Source: Islets. 2021 Dec 30;14(1):58–68. doi: 10.1080/19382014.2021.2016028 (PMC8726731; doi:10.1080/19382014.2021.2016028)
Supplement: Supplemental Material [file KISL_A_2016028_SM9073.pdf]

Table 1: Mouse qRT-PCR Primer Pairs

Gene	Forward	Reverse
Insulin I	*CCA GCT ATA ATC AGA GAC CA*	*CCA GGT GGG GAC CAC AAA GA*
Insulin II	*GGC TTC TTC TAC ACA CCC AT*	*CCA AGG TCT GAA GGT CAC CT*
MafA	*CCT GTA GAG GAA GCC GAG GAA*	*CCT CCC CCA GTC GAG TAT AGC*
Pdx1	*CGG CTG AGC AAG CTA AGG TT*	*TGG AAG AAG CGC TCT CTT TGA*
Nkx6.1	*CCT CTG GAC CCG AAC TCT GA*	*GCT GCC ACC GCT CGA TT*
SSBP3	*ATG GAG CCC CAC CAC ATG AAT G*	*CTG GAA GGA GTG GAG GAA GTT C*
Ldb1	*ACT CAT GTG GAT GCC TGT GTG*	*CCC CAA CAT TTA GCC CCT AAG*
Isl1	*GCA ACC CAA CGA CAA AAC TAA*	*CCA TCA TGT CTC TCC GGA CT*
36B4	*GCG ACC TGG AAG TCC AAC TAC*	*ATC TGC TGC ATC TGC TTG G*
Gapdh	*CCT GGA GAA ACC TGC CAA GTA*	*TGG AAG AGT GGG AGT TGC TGT*

Table 2: Human qRT-PCR Primer Pairs

Gene Name	Forward	Reverse
LDB1	*CCA GAT GTT GGA TCA GCT CTC C*	*AGG TCT TGT GGC GTG ACA TGA G*
MAFA	*TGA GCG GAG AAC GGT GAT TTC TAA GG*	*GGA ACG GAG AAC CAC GTT CAA CGT A*
PDX1	*TAC TGG ATT GGC GTT GTT TGT GGC*	*AGG GAG CCT TCC AAT GTG TAT GGT*
NKX6.1	*ATT CGT TGG GGA TGA CAG AG*	*CGA GTC CTG CTT CTT CTT GG*
ISL1	*TAC AAA GTT ACC AGC CAC C*	*GGA AGT TGA GAG GAC ATT GA*
18S	*GCT TAA TTT GAC TCA ACA CGG GA*	*GCT ATC AAT CTG TCA ATC CTG TCC*

Table 3: Western Blot Primary Antibodies

Protein	Company	Catalog #	Species	Dilution
LDB1	Santa Cruz Biotechnology, Inc	sc-11198x	Rabbit	1:1000
ISL1	Developmental Studies Hybridoma Bank	39.4D5-c	Mouse	1:1000
MAFA	Novus Biologicals	NBP1–00121	Rabbit	1:1000
NKX6.1	Developmental Studies Hybridoma Bank	F55A10-c	Mouse	1:1000
β–Actin	Cell Signaling Technology	#4967S	Rabbit	1:1000

Table 4: PLA Primary Antibodies

Protein	Company	Catalog #	Species	Dilution
LDB1	Dr. Paul Love, NIH		Rabbit	1:1000
ISL1	Developmental Studies Hybridoma Bank	39.4D5-c	Mouse	1:1000
